# Nurses’ perceptions of patient safety culture measured by the Hospital Survey on Patient Safety Culture in the Gulf Cooperation Council region: A systematic review

**DOI:** 10.1016/j.ijnsa.2026.100512

**Published:** 2026-02-21

**Authors:** Majed S. Alshammari, Jane Tyerman, Idrissa Beogo, J. Craig Phillips

**Affiliations:** School of Nursing, Faculty of Health Sciences, University of Ottawa, 200 Lees Ave, Ottawa, ON K1N 6N5, Canada

**Keywords:** Patient safety, Patient safety culture, Hospital survey on patient safety culture, Nurses, Hospitals, Gulf Cooperation Council, Systematic review

## Abstract

**Background:**

Patient safety is a global priority, and nurses play a central role in its implementation and monitoring. Understanding nurses’ perceptions of patient safety culture supports refinement of safety strategies, evidence-informed policymaking, and sustained cultural change. This systematic review synthesizes studies using the Hospital Survey on Patient Safety Culture to assess nurses’ perceptions in the Gulf Cooperation Council countries: Bahrain, Kuwait, Oman, Qatar, Saudi Arabia, and the United Arab Emirates.

**Methods:**

The review was registered with PROSPERO and guided by the PRISMA framework. CINAHL, MEDLINE, and EMBASE were searched in September 2024 using predefined keywords related to nursing, patient safety, the Hospital Survey on Patient Safety Culture, and the Gulf Cooperation Council countries. Inclusion criteria were primary studies reporting nurses’ Hospital Survey on Patient Safety Culture scores in hospital settings. Covidence software was used for study screening, duplicate removal, and eligibility assessment.

**Results:**

From 54 identified records, 25 unique studies were screened, 20 underwent full-text review, and 9 met inclusion criteria. Eight studies employed the Hospital Survey on Patient Safety Culture version 1 (nurses: 2591; mean sample size: 323.88) and one study used the Hospital Survey on Patient Safety Culture version 2 (nurses: 402). Across version 1 studies, highest average composite scores were observed for Teamwork Within Units (71.92%) and Organizational Learning–Continuous Improvement (78.70%). Communication Openness (45.51%), Staffing (40.09%), Handoffs and Transitions (40.53%), and Nonpunitive Response to Error (35.42%) were lowest. Version 2 results showed similar trends: Teamwork (77%) and Organizational Learning - Continuous Improvement (72.3%) were high, while Staffing and Work Pace (46.17%) and Response to Error (39.75%) were low. The review’s findings indicated substantial variation in nurses’ perceptions across patient safety culture composites, from high ratings for teamwork to low ratings for staffing and nonpunitive error reporting.

**Conclusions:**

Patient safety culture in the Gulf Cooperation Council hospitals shows strengths in teamwork and continuous improvement, but persistent weaknesses in staffing, error reporting, and care transitions. Aligned with national healthcare visions (e.g., Saudi Vision 2030 and Oman Vision 2040), evidence-based recommendations include implementing structured handoff processes, adopting nonpunitive and confidential approaches to promote nonpunitive error reporting, and strengthening workforce strategies to improve staffing adequacy and workload management. Additionally, it may be useful to develop instruments that are based on the sociocultural realities of these societies and not adapted from other countries' instruments.

**What is already known**

• Nurses are central to implementing and monitoring patient safety.
• The Hospital Survey on Patient Safety Culture is widely used to assess safety culture in hospitals.
• The Gulf Cooperation Council’s research on the Hospital Survey on Patient Safety Culture is fragmented, limiting regional insight.

**What this paper adds**

• Synthesizes the Gulf Cooperation Council studies on nurses’ perceptions of patient safety culture.
• Identifies strong teamwork and continuous improvement as strengths.
• Highlights concerns about staffing, error reporting, and care transitions.

Alt-text: Unlabelled box dummy alt text

## 1 Patient safety in context

Patient safety is a critical priority for healthcare organizations worldwide (Atalla et al., 2025). In 2000, the Institute of Medicine in the United States (US) highlighted this issue in its influential report, To Err Is Human. Publication of this report has significantly increased public awareness of medical errors in the US (Donaldson et al., 2000). Subsequently, international interest in the examination of patient safety culture emerged (Feng et al. 2008). In response to the significant magnitude of the worldwide patient safety concern, the World Health Organization (WHO) established the World Alliance for Patient Safety in 2004, launched by WHO leadership alongside the Pan American Health Organization, UK and U.S. governmental representatives, WHO regional directors, patient advocates, and international safety experts (WHO, 2004a, 2004b). The primary objective of this alliance was to formulate fundamental principles, generate guidelines and make suggestions aimed at mitigating risks and minimizing adverse occurrences within healthcare systems (WHO, 2004a, 2004b). Furthermore, the adoption of resolution WHA72.6 on "Global action on patient safety" in May 2019 by the Seventy-second World Health Assembly reflects widespread international attention to patient safety. The resolution called upon the WHO Director-General to prioritize patient safety as a crucial strategic objective within the scope to promote universal health coverage (WHO, 2023). WHO supported the establishment of World Patient Safety Day, set to occur annually on the 17th of September. The primary aim of this initiative is to enhance public awareness and promote a global understanding of patient safety (WHO, 2023).

## 2 The role of nurses in patient safety

Nursing is a humanitarian vocation dedicated to helping the sick or injured and ensuring high standards of healthcare services. In medical facilities, nurses constitute the largest proportion of the medical staff. The nursing profession is considered the backbone of the healthcare system, bridging various health professions and playing a central role in healthcare processes. Nurses account for nearly 50 % of the global health workforce (WHO, 2025). As frontline providers of care, nurses are central to the implementation and observation of patient safety practices. Their continuous presence at the bedside and involvement in both direct patient care and interdisciplinary communication give them a unique vantage point from which to assess the safety culture within clinical settings (Hajizadeh et al., 2025; Phillips et al., 2021). Because they operate at the intersection of policy and practice, nurses are often the first to identify deviations from standard protocols, emerging safety risks, or unintended consequences of system-level decisions (Glarcher and Vaismoradi, 2024; Hajizadeh et al., 2025). Additionally, nurses’ dual role, as both implementers of institutional policy and observers of day-to-day clinical operations, positions nurses to detect and report gaps between standard safety procedures and care delivery. Their insights are indispensable for the development of safety strategies, facilitating organizational learning, and enhancing overall quality of care (Glarcher and Vaismoradi, 2024; Reyes Ramos and Costa Abos, 2024).

## 3 The regional healthcare context of the Gulf Cooperation Council

This systematic review focuses on the Gulf Cooperation Council region, which includes Bahrain, Kuwait, Oman, Qatar, Saudi Arabia, and the United Arab Emirates. The Gulf Cooperation Council member states share a common historical and political context. Additionally, deep religious and cultural connections unite the six states, and strong kinship bonds prevail among their citizens. These factors, along with geographical continuity across the Arabian Peninsula, have facilitated interactions among them and fostered similar values and characteristics. Furthermore, the Gulf Cooperation Council countries display comparable economic features due to their reliance on revenues derived from natural resources, particularly oil, to support national development and healthcare financing (Alkhamis et al., 2014; Secretariat General of the Gulf Cooperation Council, 2024). As Christie (1987, p.7) noted, unlike many international alliances that must overcome differences in language, culture, and political systems, the Gulf Cooperation Council emphasizes similarities among its members, including a shared religion (Islam), a common language (Arabic), similar systems of governance, parallel social structures rooted in extended family and tribal relationships, comparable levels of economic development, and shared geography (Al-Hamadi et al., 2007; Farag, 2017). In addition to these contextual similarities, healthcare systems across the Gulf Cooperation Council countries exhibit comparable organizational structures, regulatory environments, and workforce models, which provide a coherent basis for examining patient safety culture within hospital settings at the regional level (Alkhamis et al., 2014; WHO, 2020).

In the Gulf Cooperation Council region, nurses are the majority of healthcare professionals, playing a vital role in all aspects of patient care. In Saudi Arabia, as of 2023, the total number of nurses in the Saudi health sector reached 213,110, with only 94,021 being Saudi nationals. The remaining nurses are primarily foreign workers, predominantly from the Philippines, India, and Malaysia (Alluhidan et al., 2020; Ministry of Health, 2023; 2024). Similarly, in Oman, nurses comprise approximately 60 % of all healthcare professionals and represent most of the healthcare workforce, with many being expatriates from India, the Philippines, and Sri Lanka (Ministry of Health, 2022; Valdez et al., 2021). A similar pattern is observed in Bahrain, where nurses constitute most of the healthcare workforce, though most are Bahraini nationals (Alhourani et al., 2022; Awadhalla et al., 2018). In the United Arab Emirates, the healthcare system heavily relies on expatriate nurses, with around 96 % originating from the Philippines, India, Pakistan, Arab nations, America, and the British Commonwealth (Al-Yateem et al., 2022). In Qatar, nurses represent the largest professional workforce in the healthcare sector, with over 90 % being expatriates from the Philippines and India (Al-Harahsheh et al., 2020; MacDonald et al., 2020). Additionally, in Kuwait, nurses make up the largest segment of healthcare practitioners, totalling 22,021. The vast majority (95.4 %) are non-Kuwaiti expatriates, primarily from India, Egypt, the Philippines, and Malaysia (Aljarida, 2023; Al-Maaitah et al., 2023). This multinational workforce composition introduces additional complexity to patient safety culture, as nurses bring diverse educational backgrounds, languages, and professional norms into shared organizational environments.

## 4 Measuring patient safety culture

Patient safety culture is widely recognized as a critical determinant of healthcare quality and clinical outcomes. It refers to the shared values, beliefs, and norms within a healthcare organization that influence staff attitudes and behaviours related to patient safety. It reflects the extent to which safety-related behaviours are supported, expected, and accepted across all organizational levels (Agency for Healthcare Research and Quality [AHRQ], 2024). A strong safety culture has been associated with lower rates of adverse events such as medication errors and hospital readmissions, as well as higher levels of patient satisfaction (Alanazi et al., 2022; Alsabri et al., 2022). Key elements of patient safety culture include such factors as open communication, error reporting, teamwork, leadership support, and a non-punitive response to errors (Churruca et al., 2021; Farokhzadian et al., 2018). Assessing patient safety culture allows healthcare organizations to identify strengths and areas for improvement, monitor changes over time, and implement targeted strategies to improve safety outcomes (Camacho-Rodríguez et al., 2022; Rawas and Abou Hashish, 2023). One of the most widely used tools for this purpose is the Hospital Survey on Patient Safety Culture developed by the AHRQ (AHRQ, 2023; Vikan et al., 2023).

The Hospital Survey on Patient Safety Culture appraises organizational elements that affect patient safety culture at both the hospital-wide level and within departments (Sorra and Nieva, 2004). The Hospital Survey on Patient Safety Culture is not only psychometrically solid, but its validity and consistency have been corroborated (Sorra and Dyer, 2010; Sorra et al., 2019). The Hospital Survey on Patient Safety Culture is considered psychometrically robust, with reliability analyses reporting Cronbach’s alpha values for the composites ranging from 0.62 to 0.85, indicating moderate to strong internal consistency across most composites (Sorra and Dyer, 2010). The survey has been used in multiple investigations to assess nurses' perceptions and understandings of patient safety culture in various Gulf Cooperation Council hospitals (Rawas and Abou Hashish, 2023; Wazqar and Attallah, 2024). The Hospital Survey on Patient Safety Culture has been used in over 99 countries, including Korea, Brazil, Spain, and the Gulf Cooperation Council region, demonstrating its global relevance and adaptability (AHRQ, 2023; Alquwez et al., 2018; Reis et al., 2023). Although several tools are available for measuring safety culture, such as the Safety Attitudes Questionnaire and the Manchester Patient Safety Framework, the Hospital Survey on Patient Safety Culture remains the most widely used globally for assessing hospital-based patient safety culture. Recent evidence shows that 104 studies utilized the Hospital Survey on Patient Safety Culture, compared to 63 using the Safety Attitudes Questionnaire (Carvalho et al., 2023), while another review found that nearly half of all quantitative hospital-based studies (*n* = 312) utilized the Hospital Survey on Patient Safety Culture followed by the Safety Attitudes Questionnaire (30.7 %) (Churruca et al., 2021). Additionally, Azyabi and colleagues (2021) conducted a systematic review to evaluate patient safety culture instruments, identifying 66 studies, of which 54 (82 %) used the Hospital Survey on Patient Safety Culture.

The first version of the Hospital Survey on Patient Safety Culture was created in 2004 and is comprised of 12 composites, in addition to two overarching questions regarding the overall patient safety degree perceived by respondents in their respective units and the number of safety events reported in the past year (AHRQ, 2013). The 12 composites are Teamwork Within Units; Supervisor/Manager Expectations & Actions Promoting Patient Safety; Organizational Learning-Continuous Improvement; Management Support for Patient Safety; Overall Perceptions of Patient Safety; Feedback & Communication About Error; Communication Openness; Frequency of Events Reported; Teamwork Across Units; Staffing; Handoffs & Transitions; and Nonpunitive Response to Error.

The Hospital Survey on Patient Safety Culture-II is an updated and validated version of the original Hospital Survey on Patient Safety Culture, refined to reflect current patient safety research and feedback from healthcare users. It reduces the number of composites from 12 to 10, removing *Overall Perceptions of Patient Safety* and *Teamwork Across Units* to streamline the tool and focus on more actionable areas. The remaining composites were largely preserved, with some title revisions to better align with contemporary terminology and clinical practice. Survey items underwent a rigorous refinement process with some items unchanged (*n* = 4), added (10), others reworded for clarity (*n* = 9), and several were significantly revised (*n* = 9) or removed to eliminate redundancy (*n* = 20) (Sorra et al., 2019). The changes improved psychometric performance, with Cronbach’s alpha for revised composites ranging from 0.67 to 0.89, reflecting enhanced internal consistency reliability (AHRQ, 2019). These changes lowered survey burden, improved response quality, and better aligned the tool with contemporary safety culture frameworks (AHRQ, 2019; Sorra et al., 2021). The Hospital Survey on Patient Safety Culture-II survey composites include: Communication About Error; Communication Openness; Handoffs and Information Exchange; Hospital Management Support for Patient Safety; Organizational Learning-Continuous Improvement; Reporting Patient Safety Events; Response to Error; Staffing and Work Pace; Supervisor, Manager, or Clinical Leader Support for Patient Safety; and Teamwork (AHRQ, 2019; Sorra et al., 2021).

Notwithstanding a dearth of studies on the subject in the Gulf Cooperation Council, previous studies have indicated varying perceptions of patient safety culture among healthcare workers, including nurses. Some studies have documented generally positive attitudes, with average composite scores exceeding 60 % (Alaska and Alkutbe, 2023; Algethami et al., 2024), while others have highlighted significant areas for improvement, reporting average scores below 60 % (Al Muharraq et al., 2024).

However, there remains a notable gap in synthesizing and comparing findings across the Gulf Cooperation Council, especially in hospital-based, acute, and critical care settings. This research gap aligns with broader findings from the Arab region, of which the Gulf Cooperation Council states are a part. Zubairi et al. (2023) reported that only 2 % of global patient safety research originated from the Arab region, with limited output from most Gulf Cooperation Council countries except Saudi Arabia, and called for increased investment in patient safety research. Similarly, Elmontsri et al. (2017) noted significant variation in study settings and participant groups in Arab countries, limiting focused conclusions, particularly regarding nurses’ perceptions. Despite this, no systematic review has yet exclusively examined nurses’ perceptions of patient safety culture within hospital and acute care settings in the Gulf Cooperation Council region. Addressing this gap is essential for identifying strengths, weaknesses, and future opportunities to enhance patient safety from the perspective of those most closely involved in direct patient care.

This systematic review aims to address the question: What are nurses’ perceptions of patient safety culture, as defined by the Hospital Survey on Patient Safety Culture, in the Gulf Cooperation Council region, which includes Bahrain, Kuwait, Oman, Qatar, Saudi Arabia, and the United Arab Emirates?

This review synthesizes data from both versions of the survey, Hospital Survey on Patient Safety Culture and Hospital Survey on Patient Safety Culture-II, to capture detailed insights into the composites of patient safety measured by each version in the Gulf Cooperation Council region. It examines how patient safety culture has been integrated into healthcare settings across the Gulf Cooperation Council. The review further aims to identify common strengths, persistent challenges, and gaps in the existing evidence in order to inform evidence-based recommendations for research, practice, and policymakers to improve patient safety culture in the Gulf Cooperation Council region.

## 5 Methods

This systematic review was conducted in accordance with the Preferred Reporting Items for Systematic Reviews and Meta-Analyses (PRISMA) guidelines (PRISMA, 2021) and was registered with the PROSPERO registry (Registration ID: CRD42024596704).

### 5.1 Literature search strategy

The electronic databases CINAHL, Medline, and EMBASE were queried in September 2024. The search used keywords "Nurs*," “health professional”, “health provider”, “health practitioner”, “patient safety”, “patient safety culture”, "Bahrain," "Kuwait," "Oman," "Qatar," “Saudi Arabia”, “Kingdom of Saudi Arabia”, “United Arab Emirates”, “Hospital Survey on Patient Safety Culture”, and "HSOPSC." Boolean operators and OR were applied to effectively combine search terms (Supplementary Table 1). The search covered the period from 2004 to 2024, reflecting the timeline since the Hospital Survey on Patient Safety Culture was first developed in 2004. Because healthcare systems in the Gulf Cooperation Council region primarily operate in English, the search was limited to studies published in English.

### 5.2 Eligibility criteria

The Population, Intervention, Comparison, and Outcomes (PICO) framework was used to define inclusion and exclusion criteria for studies (Supplementary Table 2) (Schardt et al., 2007). Studies conducted in acute care settings and published in a peer-reviewed journal were included if they: (1) surveyed nurses or other health care practitioners but separately detailed nurses’ perceptions, (2) were conducted solely in a country or countries within the GGC region, and (3) were quantitative studies that used Hospital Survey on Patient Safety Culture to survey nurses or mixed-methods studies that reported Hospital Survey on Patient Safety Culture quantitative data. Studies were excluded if they (1) do not survey nurses or studies that do not detail nurses’ perceptions separately, (2) were from all other countries and regions of the world, and (3) were quantitative studies that used other instruments to measure patient safety or mixed-method studies that did not use Hospital Survey on Patient Safety Culture or did not report Hospital Survey on Patient Safety Culture quantitative data. Although mixed-methods studies were eligible if they reported Hospital Survey on Patient Safety Culture quantitative data for nurses, no mixed-methods studies met the inclusion criteria. All included studies were quantitative in design.

### 5.3 Review process

A rigorous and structured process was followed to ensure inclusion of relevant and eligible studies. Title, abstract, and full text screening were completed by two reviewers. After applying the predefined keywords, a total of 54 articles were identified across the three electronic databases, with 20 studies from EMBASE, 19 from MEDLINE, and 15 from CINAHL. Following removal of 29 duplicate studies, 25 articles remained for further evaluation.

**Fig. 1:**
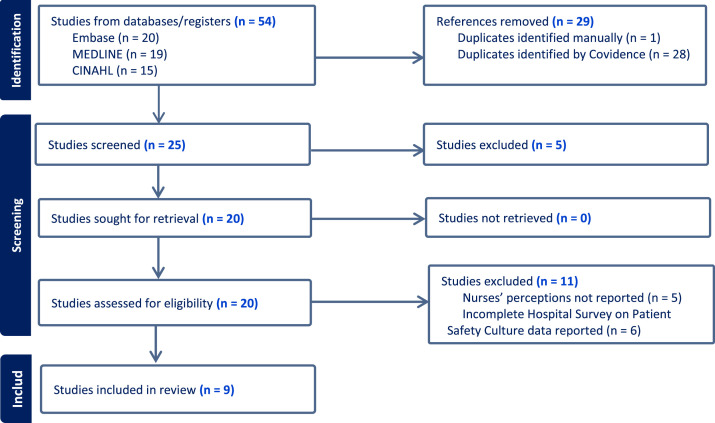
Eligibility flowchart of patient safety culture in the Gulf Cooperation Council region. Note. Flowchart adapted from the PRISMA guidelines, based on study selection data generated in Covidence systematic review software (PRISMA, 2021; Veritas Health Innovation., 2025).

During the abstract screening phase, five studies were excluded based on predefined inclusion and exclusion criteria. Subsequently, a full-text review was conducted to assess the eligibility of the remaining 20 studies, resulting in the exclusion of an additional 11 studies; five studies did not report nurses’ data separately, and six studies did not present positive response percentage scores for Hospital Survey on Patient Safety Culture composites, which precluded inclusion in the review. Of the nine studies included in the final synthesis, eight were quantitative studies focusing exclusively on nurses, and one was a quantitative study in which nurses’ perceptions were reported separately. At each stage of the review process, disagreements were resolved through discussions between two reviewers (MA, JCP) to achieve consensus. The Covidence systematic review software (Veritas Health Innovation, Melbourne, Australia) was used to organize articles, remove duplicates, and exclude ineligible studies, ensuring a transparent and reproducible process (Fig. 1) (Veritas Health Innovation., 2025).

### 5.4 Quality assessment

Quality of selected articles was independently assessed by two reviewers (MA, JCP) using the Checklist for Analytical Cross-Sectional Studies developed by the Joanna Briggs Institute (2020). This checklist includes eight criteria for evaluating study quality. To ensure relevance to the specific topic and methods of the included studies, the reviewers agreed to adapt certain criteria after careful consideration (Supplementary Table 3). The adaptations include assessing the validity and reliability of patient safety culture measurements, use of standard criteria, and clarity of the Hospital Survey on Patient Safety Culture. Additionally, the checklist examines whether the findings are appropriately summarized and reflect the collected data, ensuring both relevance and rigour for this review. Quality assessment scores for the included studies are presented in Table 1. Studies scoring below 50 %, fewer than 4 out of 8 criteria, were excluded from the final synthesis of the systematic review. As with the review process, any disagreements during quality assessment were resolved through discussions between two reviewers (MA, JCP) to achieve consensus, ensuring reliability and objectivity of the evaluation. Quality assessment and data extraction were completed in Covidence.

**Table 1:** Quality evaluation of studies: total scores (Max = 8).

Study ID	Total Score (out of 8)
Al-Awa, 2012	6
AlMuharraq, 2024	8
Alquwez, 2018	7
Ammouri, 2015	5
Wazqar, 2024	7
Aboshaiqah, 2010	8
Rawas 2023	8
AlMa'mari, 2019	6
SalehAboufour, 2022	8

Source: Adapted from the Joanna Briggs Institute (2020).

### 5.5 Data extraction

Following quality assessment of the studies, two reviewers (MA, JCP) individually extracted data from the nine eligible studies. Extracted data included the study author and year, country, participant details, sample sizes, and patient safety culture composite scores. In case of disagreements, reviewers met to discuss and resolve the issues; if unresolved, two additional reviewers (JT, IB) were available to be consulted to provide a final resolution. Consultation was not required because initial reviewers were able to resolve all disagreements and arrive at a final resolution of the issues.

## 6 Results

▒

### 6.1 Sociodemographic characteristics of samples studied

This systematic review of studies on patient safety culture in the Gulf Cooperation Council region highlights key demographic characteristics and perceptions about patient safety among nurses (Supplementary Table 4, Table 2, Table 3). The investigation included studies conducted between 2010 and 2024 that used the Hospital Survey on Patient Safety Culture to assess composites of safety culture in healthcare settings. Across multiple studies (*n* = 6), there was a notable representation of non-Arabic-speaking and expatriate nurses. For example, in one study in Saudi Arabia, most participants were non-Arabic speakers (*n* = 387, 87 %; Aboshaiqah, 2010). Similarly, the studies by Al-Awa (2012) and Alquwez (2018) in Saudi Arabia revealed a predominant proportion of Indian and Filipino nurses, with Al-Awa reporting 44.5 % Indian and 41 % Filipino participants, and Alquwez reporting 38.7 % Indian and 43.3 % Filipino nurses. In Oman, AlMa'mari (2019) reported that 81.5 % of nurses were non-Omani, while Rawas (2023) reported that 81 % of participants in Saudi Arabia were non-Saudi. Conversely, the study by AlMuharraq (2024) in Saudi Arabia reported a sample of mostly Saudi nurses (*n* = 349, 86.8 %). Gender distribution was a key characteristic across the studies (Aboshaiqah, 2010; Alquwez et al., 2018; AlMa'mari et al., 2019; Rawas and Abou Hashish, 2023; Wazqar and Attallah, 2024; AlMuharraq et al., 2024).

**Table 2:** Studies utilizing the first version of hospital survey on patient safety culture, nurses’ perspectives (*N* = 2591).

**Author, year**	Aboshaiqah, 2010	Al-Awa, 2012	Ammouri, 2015	Alquwez, 2018	Al Ma’mari, 2019	Saleh Aboufour, 2022	Rawas, 2023	Wazqar, 2024	Average*
**Country**	KSA	KSA	Oman	KSA	Oman	KSA	KSA	KSA	N/A
**sample size, Nurses (n)**	445	605	414	351	270	221	184	101	323.88
**Composite scores**									
Teamwork Within Units	70	68	83.4	85.8	94	25.4	82.95	65.84	71.92
Supervisor /Manager Expectations & Actions Promoting Patient Safety	49	51	60	54.3	59.9	76.9	72.86	46.54	58.81
Organizational Learning-Continuous Improvement	82	74	81.1	83.3	86.3	48.9	81.88	92.08	78.70
Management Support for Patient Safety	90	61	25.2	60.3	65.9	18.1	70.15	66.01	57.08
Feedback & Communication About Error	67	58	68.7	56.3	77.7	49.3	81.25	76.57	66.85
Communication Openness	36	36	49.7	37.4	45.6	76.5	55.80	27.06	45.51
Frequency of Events Reported	61	57	58.8	20.3	61.7	68.3	73.82	75.04	59.50
Teamwork Across Units	55	51	66.1	59.3	50.2	69.3	67.29	69.31	60.94
Staffing	54	15	27	21.1	57	82	40.08	24.50	40.09
Handoffs & Transitions	22	47	57.7	46.1	15.3	25.3	39.06	71.79	40.53
Nonpunitive Response to Error	49	16	21.4	16.6	54.8	59.2	43.89	22.44	35.42
Overall Perceptions of Patient Safety	52	45	50.7	48.8	61.4	85.6	59.00	69.31	58.98

⁎ The percentage is derived from the total sample size for the studies.

**Table 3:** Study using hospital survey on Patient Safety Culture Version II.

**Author, year**	Al Muharraq, 2024
**Country**	KSA
**Sample size, nurses**	402
**Composite scores**	
Teamwork	77
Staffing and Work Pace	46.17
Organizational Learning-Continuous Improvement	72.3
Response to Error	39.75
Supervisor, Manager, or Clinical Leader Support for Patient Safety	59.56
Communication About Error	69.36
Communication Openness	55.75
Reporting Patient Safety Events	59.45
Hospital Management Support for Patient Safety	61.1
Handoffs and Information Exchange	58.3

Female nurses were overwhelmingly represented in most studies (*n* = 1493, 85.22 %), with proportions ranging from 82.6 % in Rawas (2023) to 91.5 % in Alquwez (2018). In Oman, Ammouri (2015) reported 89.6 % female nurses, while in Saudi Arabia, AlMuharraq (2024) reported 84.1 % female participants. Wazqar (2024) in Saudi Arabia was unique in reporting a notable percentage of male nurses, with 39.6 % of participants being male and 60.4 % female.

The mean age of nurses across studies was relatively consistent, ranging from 28.63 years in Alquwez (2018) to 35.11 years in Aboshaiqah (2010). Studies in Oman, such as Ammouri (2015) and AlMa'mari (2019), reported mean ages of 35 years and 33.06 years, respectively. In Saudi Arabia, Rawas (2023), Wazqar (2024), and AlMuharraq (2024) reported mean ages of 32.5 years, 31 years, and 30.8 years, respectively. Work experience was another notable demographic feature.

In Aboshaiqah’s (2010) study, 80.9 % of nurses had more than six years of experience, while Wazqar (2024) reported 63.4 % of participants with similar experience. On the other hand, Alquwez (2018) and Rawas (2023) reported that 72.1 % and 57.1 % of participants, respectively, had less than six years of nursing experience.

In terms of educational qualifications, most nurses across the studies held a bachelor’s degree. For instance, Aboshaiqah (2010) reported 85.4 % of nurses with a bachelor’s degree, and Alquwez (2018) and Rawas (2023) reported 88.9 % and 90.8 %, respectively. However, diploma holders were significant in studies from Oman and Saudi Arabia (*n* = 271, 20.72 %) (Aboshaiqah, 2010; Alquwez et al., 2018; AlMa'mari et al., 2019; Rawas and Abou Hashish, 2023; Wazqar and Attallah, 2024; AlMuharraq et al., 2024). For example, Ammouri (2015) reported that 65.4 % of nurses held a diploma, and AlMa'mari (2019) found that 33.3 % of nurses had a diploma. The study by AlMuharraq (2024) also reported 34.3 % diploma holders and 5.7 % holding master’s degrees. Work hours varied across studies, with most nurses working 40–59 h per week. For instance, Alquwez (2018) reported that 82.1 % of nurses worked 40–59 h weekly, while Rawas (2023) indicated that 67.4 % worked similar hours. Wazqar (2024) reported that 79.2 % of nurses worked >40 h weekly.

### 6.2 Patient safety culture composites scores from reviewed studies

Two versions of the Hospital Survey on Patient Safety Culture survey were used to evaluate nurses' perceptions of various composites of patient safety culture in the Gulf Cooperation Council region. Studies employing the first version of the Hospital Survey on Patient Safety Culture (Table 2) included six investigations conducted in Saudi Arabia and two in Oman, with sample sizes ranging from 101 to 605 nurses, an average sample size of 323.88, and a total sample size across studies of 2591. Across these studies, the composite scores exhibited considerable variation.

For the composite of Teamwork Within Units, scores ranged from 25.4 % (SalehAboufour and Subbarayalu, 2022) to 94 % (AlMa'mari et al., 2019), with many studies reporting relatively high values, indicating a generally positive perception of collaboration within individual units; across all studies, the average score was 71.92 %.

Supervisor/Manager Expectations and Actions Promoting Patient Safety showed moderate variability, with scores between 46.54 % (Wazqar and Attallah, 2024) and 76.9 % (SalehAboufour and Subbarayalu, 2022), reflecting mixed perceptions of supervisory support for safety initiatives, with an average score of 58.81 % across all studies.

Organizational Learning-Continuous Improvement was consistently one of the higher-scoring s, with values ranging from 48.9 % (SalehAboufour and Subbarayalu, 2022) to 92.08 % (Wazqar and Attallah, 2024), highlighting an emphasis on improvement efforts in many settings; across all studies, the average score was 78.70 %.

Management Support for Patient Safety revealed significant variability, with scores spanning from a low of 18.1 % (SalehAboufour and Subbarayalu, 2022) to a high of 90 % (Aboshaiqah, 2010), suggesting inconsistent perceptions of managerial involvement across institutions, with an average score of 57.08 % across all studies.

Feedback and Communication About Error had a range of 49.3 % (SalehAboufour and Subbarayalu, 2022) to 81.25 % (Rawas and Abou Hashish, 2023), indicating some strengths in sharing information about mistakes but also showing areas for potential growth. The average score across all studies was 66.85 %.

Communication Openness was one of the lowest-scoring composites, with values between 27.06 % (Wazqar and Attallah, 2024) and 76.5 % (SalehAboufour and Subbarayalu, 2022), revealing ongoing challenges in fostering open discussions about patient safety; across all studies, the average score was 45.51 %.

The Frequency of Events Reported composite showed variability as well, with scores from 20.3 % (Alquwez et al., 2018) to 75.04 % (Wazqar and Attallah, 2024), reflecting differences in the reporting culture across institutions, with an average score of 59.50 % across all studies.

Teamwork Across Units scored between 50.2 % (AlMa'mari et al., 2019) and 69.31 % (Wazqar and Attallah, 2024), indicating moderate collaboration across institution units. The average score across all studies was 60.94 %.

Staffing emerged as a significant challenge, with scores ranging from 15 % (Al-Awa et al., 2012) to 82 % (SalehAboufour and Subbarayalu, 2022), and an average of 40.09 %, highlighting concerns about resource adequacy.

Similarly, Handoffs and Transitions scored poorly in most studies, ranging from 15.3 % (AlMa'mari et al., 2019) to 71.79 % (Wazqar and Attallah, 2024), illustrating persistent issues in ensuring safe transitions of care; across all studies, the average score was 40.53 %.

Nonpunitive Response to Error was low in all studies ranging from 16 % (Al-Awa et al., 2012) to 59.2 % (SalehAboufour and Subbarayalu, 2022), potentially inferring institutions struggle with creating environments where staff feel safe to report errors without fear of blame or reprisal as the average score across all studies was 35.42 %.

Lastly, Overall Perceptions of Patient Safety scored between 45 % (Al-Awa et al., 2012) and 85.6 % (SalehAboufour and Subbarayalu, 2022), reflecting varying overall assessments of safety within institutions, with an average score of 58.98 % across all studies.

On the other hand, one study (Table 3) utilized the second version of Hospital Survey on Patient Safety Culture to evaluate patient safety culture in KSA, with a sample of 402 nurses (AlMuharraq, 2024). This study reported a strong score for Teamwork (77 %), reflecting positive collaboration among staff. However, Staffing and Work Pace scored 46.17 %, indicating concerns about workload and resources. Organizational Learning-Continuous Improvement achieved a score of 72.3 %, underscoring ongoing efforts to enhance safety practices. The Response to Error composite scored 39.75 %, highlighting the need for improvement in addressing and learning from errors. Supervisor, Manager, or Clinical Leader Support for Patient Safety scored 59.56 %, reflecting moderate leadership involvement in promoting safety. Communication About Error was reported at 69.36 %, suggesting relatively favourable practices in addressing errors. Communication Openness scored 55.75 %, indicating progress but with room for further improvement in fostering transparent communication. Reporting Patient Safety Events received a score of 59.45 %, pointing to moderate levels of event reporting. Hospital Management Support for Patient Safety achieved 61.1 %, showing average perceptions of management’s commitment to safety. Finally, Handoffs and Information Exchange scored 58.3 %, reflecting moderate effectiveness in transitions of care and information sharing (AlMuharraq, 2024). Across all the studies, these findings provide a more comprehensive view of patient safety culture within healthcare institutions in the Gulf Cooperation Council region, with notable strengths and areas requiring significant improvement across different composites, which will be explored in the discussion section.

## 7 Discussion

This systematic review assessed patient safety culture among nurses in the Gulf Cooperation Council region using both the Hospital Survey on Patient Safety Culture and Hospital Survey on Patient Safety Culture -II. The findings highlighted demographic patterns, perceptions of patient safety culture, and key areas for improvement. Although all studies included in this systematic review were conducted in Saudi Arabia and Oman, a predominant representation of expatriate nurses across many studies of this review, particularly from India and the Philippines, was observed. Gender distribution also revealed a predominance of female nurses across studies, with variations in male representation in specific contexts. Nurses’ perceptions of patient safety culture varied significantly across different composites. Teamwork Within Units was generally rated positively, whereas Staffing, Nonpunitive Response to Error, and Handoffs & Transitions emerged as key areas of concern, with consistently low scores across studies. These findings may be influenced by the global nursing shortage (WHO, 2025), high nurse turnover rates (Ren et al., 2024), and the persistence of blame-oriented cultures within clinical practice settings (Alaska and Alkutbe, 2023). Management Support for Patient Safety demonstrated significant variability, indicating inconsistent leadership commitment to safety culture. These findings align with global trends, including in Arab countries, where teamwork within units is typically a strength, while staffing and error-reporting cultures remain challenges (Al Muharraq et al., 2024; Elmontsri et al., 2017).

In addition, the expatriate profile of the nursing workforce is important for interpreting patient safety culture findings in Gulf Cooperation Council hospitals. Contemporary evidence from the United Arab Emirates suggests that language-related communication barriers experienced by expatriate (non-Arabic-speaking) healthcare practitioners may affect the quality and safety of care delivery, particularly in day-to-day clinical communication and information exchange (Al-Yateem et al., 2022). Evidence from large-scale patient safety monitoring also supports the broader relevance of language barriers to patient harm risk in hospital settings (Canadian Institute for Health Information, 2024).

However, although some of the included studies reported the nationality composition of samples, none of the included studies reported Hospital Survey on Patient Safety Culture composite results stratified by nurse nationality or language group. Accordingly, it was not possible for this review to determine whether expatriate and national nurses perceived patient safety culture differently across Hospital Survey on Patient Safety Culture composites. This represents an important evidence gap and a priority for future patient safety culture research in the Gulf Cooperation Council.

When compared to international findings, the patient safety culture composites assessed in the Gulf Cooperation Council exhibit both similarities and unique challenges. Nurses in Iran reported low patient safety culture scores, with Nonpunitive Response to Error, Staffing, and Handoffs & Transitions being among the lowest-rated composites, similar to the Gulf Cooperation Council region (Kakemam et al., 2021). Likewise, a multicountry European analysis encompassing Germany, the Netherlands, Estonia, and the United Kingdom, found that handoffs & transitions, and staffing consistently underperformed across several health systems (Sharp et al., 2019). In Portugal, multiple composites were perceived negatively; four were rated below 36 %, including Staffing, Nonpunitive Response to Error, Management Support for Patient Safety, and Frequency of Events Reported (da Costa Brás et al., 2023). Conversely, findings from a U.S. study indicated that all patient safety culture composites were rated more favorably, with scores exceeding 55 % (Tyler et al., 2024), although a prior study had identified Handoffs & Transitions and Nonpunitive Response to Error as areas of concern (Famolaro et al., 2018). Notably, nurses in Jordan reported a positive patient safety culture and rated Communication Openness and Handoffs & Transitions as a strength, opposite to the Gulf Cooperation Council region (Oweidat et al., 2024). The findings of this review suggest that while continuous improvement and teamwork within units are prioritized across healthcare systems, staffing shortages and punitive cultures hinder progress in patient safety culture. In Saudi Arabia, making the culture of healthcare safer for patients and healthcare providers is a priority that aligns with Saudi Vision 2030, which emphasizes improving access to healthcare, modernizing facilities and equipment, enhancing private sector investment, and improving the value of healthcare (Vision 2030, 2025). Additionally, Oman Vision 2040 aspires to a leading healthcare system with international standards (Ministry of Health, 2025), the fifth pillar of Kuwait Vision 2035 focuses on high-quality healthcare (Ministry of Foreign Affairs, 2025), Qatar Vision 2030 aims to create a world-class, patient-centered healthcare system, and the United Arab Emirates' Vision 2031 prioritizes the availability of high-quality healthcare services for all citizens (We the UAE 2031, 2025). Additionally, Bahrain Vision 2030 aims to elevate health standards and improve the quality of life for all residents (Ministry of Health, 2025).

### 7.1 Hospital survey on patient safety culture

The high average composites in Hospital Survey on Patient Safety Culture (75 % and above) included Organizational Learning & Continuous Improvement (78.70 %) (Westat et al., 2014). This composite consistently showed stronger performance across studies, aligning more closely with findings in Jordan (Oweidat et al., 2024) and Palestine (Zabin et al., 2022) studies. The high scores in this composite suggest that hospitals in the Gulf Cooperation Council region are actively engaging in continuous improvement efforts, which is a key strength in patient safety culture (Albalawi et al., 2020; Moafimadani et al., 2020).

The medium average composites (51 % to 74 %) included Teamwork Within Units (71.92 %), Supervisor/Manager Expectations & Actions Promoting Patient Safety (58.81 %), Management Support for Patient Safety (57.08 %), Feedback & Communication About Error (66.85), Frequency of Events Reported (59.50 %), Teamwork Across Units (60.94 %), and Overall Perceptions of Patient Safety (58.98 %) (Westat et al. 2014). These composites indicated moderate levels of implementation and effectiveness. Latin American hospitals, Sweden, Spain, and Croatia scored lower in management support except for Hungary, with similar results, while Palestine and Jordan scored higher than Gulf Cooperation Council hospitals (Camacho-Rodríguez et al., 2022; Granel-Giménez et al., 2022; Oweidat et al., 2024; Zabin et al., 2022). The moderate performance in feedback and communication about errors and Frequency of Events Reported in the Gulf Cooperation Council may be influenced by a lack of structured feedback systems and an entrenched culture of blame (Abdurabuh et al., 2024; Kaud et al., 2022). Additionally, a lack of management support was observed in some Gulf Cooperation Council hospitals, leading to reduced reporting of adverse events and lower Overall Perceptions of Patient Safety (Alaska and Alkutbe, 2023). This may reflect the dominant top-down management structure in many healthcare systems, where limited participatory leadership and restricted communication channels can hinder cross-unit collaboration and discourage frontline staff from actively engaging in safety initiatives (Essex et al., 2023; Kanaris, 2023). Encouraging effective communication, learning from previous errors and giving feedback have been linked to better patient safety outcomes (Famolaro et al., 2018; Labrague et al., 2020). Additionally, supervisors, managers, and healthcare providers should work collaboratively to promote teamwork within and across units, addressing patient safety issues and fostering a more supportive safety culture (Hayashi et al., 2020; Rosen et al., 2018).

The low average composites (50 % and below) included Staffing (40.09 %), Nonpunitive Response to Error (35.42 %), Handoffs & Transitions (40.53 %), and Communication Openness (45.51 %; Westat et al. 2014). These composites require urgent improvement, as they are consistently rated poorly among Gulf Cooperation Council nurses, highlighting challenges in maintaining adequate workforce levels and ensuring high-quality healthcare. The low ratings of these composites align with findings from studies in Iran and Latin American hospitals (Camacho-Rodríguez et al., 2022; Kakemam et al., 2021). Similarly, nurses in European countries such as Spain, Hungary, and Croatia rated these composites poorly – except for Handoffs & Transitions – confirming that blame culture, poor communication, and nursing shortages are global issues (Granel-Giménez et al., 2022). In contrast, nurses in Palestine rated these composites positively, with even higher scores reported in Jordan (Oweidat et al., 2024; Zabin et al., 2022). The stronger ratings in Jordan may be attributed to a greater emphasis on transparent communication, nonpunitive environments, and hospital management support, all of which have been shown to enhance patient safety culture (Abdurabuh et al., 2024; Daheshi et al., 2023).

### 7.2 Hospital Survey on Patient SafetyCulture-ll

A similar comparison was conducted using the second version of the Hospital Survey on Patient Safety Culture, in which a single study assessed 10 patient safety culture composites. High-average composite scores (75 % and above) included Teamwork (77 %) (Westat et al., 2014). These findings were comparable to results from Brazil, China, and India using Hospital Survey on Patient Safety Culture-ll (Aileen et al., 2024; Reis et al., 2023; Wu et al., 2022). Although teamwork was rated positively by nurses in this Gulf Cooperation Council study, similarly high ratings were observed in Brazil, China, and India (Al Muharraq et al., 2024). Additionally, the Chinese findings from Hospital Survey on Patient Safety Culture-II demonstrated higher scores in Teamwork compared to other studies, along with superior ratings in Supervisor or Clinical Leader Support for Patient Safety and Hospital Management Support. Since the healthcare field relies on collaboration among diverse specialists to deliver effective care, teamwork plays a crucial role in shaping patient safety culture (Schot et al., 2020). Moreover, fostering a strong safety culture through strategies such as promoting teamwork and effective leadership has been shown to enhance healthcare outcomes and reduce adverse clinical events (Daheshi et al., 2023; Labrague et al., 2020). These findings suggest that structured leadership programs in Chinese hospitals could serve as a model for improving patient safety culture in Gulf Cooperation Council institutions.

Medium-average composite scores (51 % to 74 %) were observed in Organizational Learning & Continuous Improvement (72.3 %), Supervisor, Manager, or Clinical Leader Support for Patient Safety (59.56 %), Communication About Error (69.36 %), Communication Openness (55.75 %), Reporting Patient Safety Events (59.45 %), Hospital Management Support for Patient Safety (61.1 %), and Handoffs and Information Exchange (58.3 %) (Westat et al., 2014). These results align with findings from India (Aileen et al., 2024) and Brazil (Reis et al., 2023), where similar levels of communication and leadership involvement were reported. Although Chinese nurses faced some challenges in reporting patient safety events, they demonstrated stronger performance in communication about errors, leadership, and management support (Wu et al., 2022). This highlights the need for greater efforts to enhance communication and leadership engagement in the Gulf Cooperation Council region. In contrast, Organizational Learning & Continuous Improvement in this Gulf Cooperation Council study was rated higher than in China, which may be attributed to the availability of dedicated educational spaces in Gulf Cooperation Council hospitals that support organizational learning (Alkhazim et al., 2015).

The low-average composite scores (50 % and below) included Staffing and Workplace (46.17 %) and Response to Error (39.75 %; Westat et al., 2014). When comparing these results with findings from Brazil, India, and China, the Gulf Cooperation Council study's scores were lower than those reported in the other studies, except for the Staffing and Workplace rating in India (Aileen et al., 2024; Reis et al., 2023; Wu et al., 2022). Although Gulf Cooperation Council countries employ foreign workers to address healthcare workforce shortages, including in nursing, staffing issues remain a persistent challenge (Al Muharraq et al., 2024). These staffing shortages directly impact patient safety and the overall quality of healthcare services (Ali et al., 2022). Additionally, previous studies have consistently identified Staffing and Response to Error as among the lowest-rated composites by nurses, including those in the Gulf Cooperation Council region (Alaska and Alkutbe, 2023; El-Shabrawy et al., 2017). This may be attributed to a prevailing blame-and-shame culture, fear of punishment, high stress levels, job dissatisfaction, and burnout factors that are exacerbated by staffing shortages (Azyabi et al., 2022; He et al., 2023). Therefore, fostering a non-punitive response culture and maintaining adequate staffing levels are essential for improving patient safety culture (Falcone et al., 2022).

### 7.3 Implications

This review synthesized findings from both versions of the Hospital Survey on Patient Safety Culture, providing a more comprehensive assessment of patient safety culture composites in the Gulf Cooperation Council region than a single study can provide. By systematically analyzing studies from 2010 to 2024, this review captured trends and variations in perceptions over time. The use of PRISMA guidelines ensured methodological rigour, while Covidence software facilitated transparent data management. Additionally, the focus on nurses’ perceptions allowed for a targeted analysis of frontline healthcare providers who play a critical role in patient safety.

The findings of this review have significant implications for policy and practice in the Gulf Cooperation Council region. First, the consistently low composites for Staffing, Nonpunitive Response to Error, and Handoffs & Transitions suggest priority targets for organizational interventions and health system planning. In international Hospital Survey on Patient Safety Culture-II benchmarking, staffing has been repeatedly identified among the lowest-scoring composite measures, indicating that this domain represents a persistent vulnerability that is sensitive to system pressures and workforce capacity (Tyler et al., 2024). Accordingly, Gulf Cooperation Council leadership strategies should prioritize workforce retention, safe staffing models, and workload management to reduce time pressure and support safer care delivery.

Second, strengthening a learning environment that supports reporting without blame is essential. The consistently low nonpunitive response composites indicate a need to build “learning-oriented” reporting systems and leadership behaviours that focus on improvement rather than punishment. These changes are also likely to strengthen event reporting and feedback mechanisms, which were only moderate in the included studies.

Third, the high representation of expatriate nurses highlights the need to strengthen communication and safety practices within multicultural and multilingual clinical environments. Evidence from the UAE indicates that expatriate clinicians experience language-related communication barriers in routine care delivery, which has implications for patient safety and quality (Al-Yateem et al., 2022).

Fourth, Tailored interventions, such as structured handoff tools, communication training, and accessible interpretation support, may be particularly relevant to improving handoffs, communication openness, and event reporting in Gulf Cooperation Council hospitals.

Finally, future research should explore the longitudinal impact of national patient safety initiatives, such as those under Saudi Vision 2030 and other Gulf Cooperation Council national healthcare visions, on patient safety culture improvement. The Saudi Health Sector Transformation Program explicitly aims to improve healthcare access and quality, providing a strategic opportunity to link routine safety culture measurement to targeted improvement initiatives in hospital settings (Vision 2030, 2025).

Future studies ought to examine the effectiveness of confidential incident reporting systems, culturally responsive leadership development, and routine safety culture assessment to guide targeted interventions.

### 7.4 Limitations

Several limitations should be acknowledged. First, this review relied on studies from only three databases and reported nurses' findings separately, which may have excluded other relevant perspectives from additional studies. Although English is the official language used for healthcare scholarship and institutional reporting in Gulf Cooperation Council settings, relevant evidence indexed elsewhere or disseminated through local outlets may not have been captured.

Second, all included studies were quantitative and relied on self-reported survey data, which may be subject to response bias and limit causal inference. The absence of qualitative or mixed-methods studies reduces the ability to explain why certain composites were low and how organizational dynamics influence reporting behaviour.

Third, the evidence base was geographically concentrated: included studies were conducted in Saudi Arabia and Oman, which limits generalizability to other Gulf Cooperation Council countries. This is a major contextual limitation and underscores the need for expanded research across Bahrain, Kuwait, Qatar, and the United Arab Emirates. In addition, the focus on Gulf Cooperation Council countries and the inclusion of studies using the Hospital Survey on Patient Safety Culture instrument may influence the extent to which findings can be generalized to different regions or studies using alternative measurement tools. However, this methodological approach enhanced consistency and comparability of composite scores across studies, supporting a more coherent synthesis of patient safety culture findings within similar healthcare contexts.

Fourth, while expatriate nurses were prominent in several samples, the primary studies did not consistently provide Hospital Survey on Patient Safety Culture outcomes stratified by nationality, language, or country of training. As a result, the review cannot determine whether differences in perceptions exist between expatriate and national nurses or among expatriate subgroups. Future Gulf Cooperation Council studies should incorporate standardized reporting of Hospital Survey on Patient Safety Culture composites by nationality/language group and examine multilingual communication factors that may shape safety culture.

Finally, this review included both the Hospital Survey on Patient Safety Culture version 1 and Hospital Survey on Patient Safety Culture-II. Although comparability concerns were mitigated by analyzing findings separately by instrument version, differences in composite structure mean that direct pooling across versions remains inappropriate.

## 8 Conclusion

This systematic review underscores the complexity of patient safety culture in Gulf Cooperation Council hospital settings, highlighting both strengths and areas needing improvement. Across included studies, teamwork and organizational learning–continuous improvement were relatively strong, suggesting a foundation for improvement efforts. However, four composites require targeted action: staffing, handoffs and transitions, communication openness, and non-punitive response to error. These findings provide an evidence base for prioritizing workforce strategies, safer transition processes, and leadership practices that support learning from errors without blame. Evidence-based recommendations derived from the review’s composite patterns include: (1) strengthening staffing capacity and managing work pace to support safe care delivery; (2) implementing and monitoring structured handoff processes; (3) adopting nonpunitive and confidential reporting approaches to improve response to error; and (4) investing in communication and teamwork interventions tailored to multilingual, multicultural nursing teams. In parallel, future research should expand coverage across all Gulf Cooperation Council countries and report safety culture outcomes stratified by nurse nationality/language to better understand safety culture in expatriate-majority hospital workforces. These actions align with Gulf Cooperation Council health sector transformation agendas and provide a practical pathway for improving patient safety culture based on the empirical patterns identified in this review. Additionally, it may be useful to develop instruments that are based on the sociocultural realities of these societies and not adapted from other countries' instruments.

## Data availability statement

The full search strategy used in this study is available in a public repository and will be provided upon acceptance of the manuscript.

## Funding

This work has received no funding.

## CRediT authorship contribution statement

**Majed S. Alshammari:** Writing – review & editing, Writing – original draft, Visualization, Validation, Software, Project administration, Methodology, Investigation, Formal analysis, Data curation, Conceptualization. **Jane Tyerman:** Writing – review & editing, Visualization, Validation, Methodology, Conceptualization. **Idrissa Beogo:** Writing – review & editing, Visualization, Validation, Investigation, Conceptualization. **J. Craig Phillips:** Writing – review & editing, Writing – original draft, Visualization, Validation, Supervision, Software, Resources, Project administration, Methodology, Investigation, Formal analysis, Data curation, Conceptualization.

## Declaration of competing interest

The authors declare that they have no known competing financial interests or personal relationships that could have appeared to influence the work reported in this paper.

## Appendix Supplementary materials

Image, application 1

Image, application 2

Image, application 3

Image, application 4
