# Supplementary material for: Nurses’ perceptions of patient safety culture measured by the Hospital Survey on Patient Safety Culture in the Gulf Cooperation Council region: A systematic review
Source: Int J Nurs Stud Adv. 2026 Feb 21;10:100512. doi: 10.1016/j.ijnsa.2026.100512 (PMC12969805; doi:10.1016/j.ijnsa.2026.100512)
Supplement: Supplementary file 1 [file mmc1.docx]

**Supplementary Table 1.** Search Strategy

**Covidence – Patient Safety Culture in GCC – September 19, 2024**

**September 19, 2024: importation by databases**

Total records: 54

**Medline = 19**

**CINAHL = 15**

**Embase = 20**

Database: Ovid MEDLINE(R) ALL <1946 to September 19, 2024>

Search Strategy:

--------------------------------------------------------------------------------

1 (Nurs* or "Health professional*" or "Health Provider*" or "Health practitioner*").mp. [mp=title, book title, abstract, original title, name of substance word, subject heading word, floating sub-heading word, keyword heading word, organism supplementary concept word, protocol supplementary concept word, rare disease supplementary concept word, unique identifier, synonyms, population supplementary concept word, anatomy supplementary concept word] (917291)

2 ("Patient safety" or "Patient safety culture").mp. [mp=title, book title, abstract, original title, name of substance word, subject heading word, floating sub-heading word, keyword heading word, organism supplementary concept word, protocol supplementary concept word, rare disease supplementary concept word, unique identifier, synonyms, population supplementary concept word, anatomy supplementary concept word] (60820)

3 (Bahrain or Kuwait or Oman or Qatar or "Saudi Arabia" or "Kingdom of Saudi Arabia" or "United Arab Emirates").mp. [mp=title, book title, abstract, original title, name of substance word, subject heading word, floating sub-heading word, keyword heading word, organism supplementary concept word, protocol supplementary concept word, rare disease supplementary concept word, unique identifier, synonyms, population supplementary concept word, anatomy supplementary concept word] (53885)

4 ("Hospital survey on patient safety culture" or "Hospital survey of patient safety culture" or HSOPSC).mp. [mp=title, book title, abstract, original title, name of substance word, subject heading word, floating sub-heading word, keyword heading word, organism supplementary concept word, protocol supplementary concept word, rare disease supplementary concept word, unique identifier, synonyms, population supplementary concept word, anatomy supplementary concept word] (437)

5 1 and 2 and 3 and 4 (19)

*************************************

Database: CINAHL ALL <1946 to September 19, 2024>

Search Strategy:

--------------------------------------------------------------------------------

( Nurs* OR “Health professional*” OR “Health Provider*” OR “Health practitioner*” ) AND ( “Patient safety” OR “Patient safety culture” ) AND ( Bahrain OR Kuwait OR Oman OR Qatar OR “Saudi Arabia” OR “Kingdom of Saudi Arabia” OR “United Arab Emirates” ) AND ( “Hospital survey on patient safety culture” OR “Hospital survey of patient safety culture” OR HSOPSC )

*************************************

Database: Embase <1974 to 2024 September 20>

Search Strategy:

--------------------------------------------------------------------------------

1 (Nurs* or "Health professional*" or "Health Provider*" or "Health practitioner*").mp. [mp=title, abstract, heading word, drug trade name, original title, device manufacturer, drug manufacturer, device trade name, keyword heading word, floating subheading word, candidate term word] (1057839)

2 ("Patient safety" or "Patient safety culture").mp. [mp=title, abstract, heading word, drug trade name, original title, device manufacturer, drug manufacturer, device trade name, keyword heading word, floating subheading word, candidate term word] (188209)

3 (Bahrain or Kuwait or Oman or Qatar or "Saudi Arabia" or "Kingdom of Saudi Arabia" or "United Arab Emirates").mp. [mp=title, abstract, heading word, drug trade name, original title, device manufacturer, drug manufacturer, device trade name, keyword heading word, floating subheading word, candidate term word] (64402)

4 ("Hospital survey on patient safety culture" or "Hospital survey of patient safety culture" or HSOPSC).mp. [mp=title, abstract, heading word, drug trade name, original title, device manufacturer, drug manufacturer, device trade name, keyword heading word, floating subheading word, candidate term word] (460)

5 1 and 2 and 3 and 4 (20)
