# Supplementary material for: Nurses’ perceptions of patient safety culture measured by the Hospital Survey on Patient Safety Culture in the Gulf Cooperation Council region: A systematic review
Source: Int J Nurs Stud Adv. 2026 Feb 21;10:100512. doi: 10.1016/j.ijnsa.2026.100512 (PMC12969805; doi:10.1016/j.ijnsa.2026.100512)
Supplement: Supplementary file 2 [file mmc2.docx]

PICO framework

| **PICO** | **Inclusion Criteria** | **Exclusion Criteria** |
| --- | --- | --- |
| Population | - Articles that surveyed nurses. - Articles that surveyed others, but detailed nurses’ perceptions separately. | - Articles that do not survey nurses - Articles that do not detail nurses’ perceptions separately. |
| Intervention | - -- | - Articles that do not focus on patient safety culture. |
| Comparison | - Studies that only have been conducted in a country or countries of the GGC region | - Studies from all other countries and regions of the world will be excluded |
| Outcomes | - Quantitative studies that used Hospital survey on patient safety culture (HSOPSC) to survey nurses. - Mixed methods studies that used HSOPSC will be included if the HSOPSC quantitative data is reported | - Other instruments used to measure patient safety culture in quantitative studies will be excluded. - Mixed methods studies that did not use HSOPSC or the HSOPSC quantitative data is not reported will be excluded. |

Schardt, C., Adams, M. B., Owens, T., Keitz, S., & Fontelo, P. (2007). Utilization of the PICO framework to improve searching PubMed for clinical questions. *BMC medical informatics and decision making*, *7*, 16. https://doi.org/10.1186/1472-6947-7-16
