# Supplementary material for: Nurses’ perceptions of patient safety culture measured by the Hospital Survey on Patient Safety Culture in the Gulf Cooperation Council region: A systematic review
Source: Int J Nurs Stud Adv. 2026 Feb 21;10:100512. doi: 10.1016/j.ijnsa.2026.100512 (PMC12969805; doi:10.1016/j.ijnsa.2026.100512)
Supplement: Supplementary file 3 [file mmc3.docx]

Quality assessment template

| Question | Adjusted to |
| --- | --- |
| Were the criteria for inclusion in the sample clearly defined? | N/A |
| Were the study subjects and the setting described in detail? | N/A |
| Was the exposure measured in a valid and reliable way? | Was the patient safety culture assessed in a valid and reliable way? |
| Were objective, standard criteria used for measurement of the condition? | Were objective, standard criteria used for the measurement of patient safety culture? |
| Were confounding factors identified? | Was the survey tool clearly described and appropriately adapted for the study context? |
| Were strategies to deal with confounding factors stated? | Were the findings appropriately summarized and reflective of the data collected? |
| Were the outcomes measured validly and reliably? | N/A |
| Was appropriate statistical analysis used? | N/A |

Source: Adapted from the Joanna Briggs Institute (2020).

Joanna Briggs Institute. (2020). Checklist for Analytical Cross-Sectional Studies. Retrieved from <https://jbi.global/critical-appraisal-tools>
