# Supplementary material for: Nurses’ perceptions of patient safety culture measured by the Hospital Survey on Patient Safety Culture in the Gulf Cooperation Council region: A systematic review
Source: Int J Nurs Stud Adv. 2026 Feb 21;10:100512. doi: 10.1016/j.ijnsa.2026.100512 (PMC12969805; doi:10.1016/j.ijnsa.2026.100512)
Supplement: Supplementary file 4 [file mmc4.docx]

| Author/  Year/  Country | Aboshaiqah  2010  Ph.d dissertation  **KSA** | Al-Awa  2012  **KSA** | Ammouri  2015  **Oman** | Alquwez  2018  **KSA** | AlMa'mari  2019  **Oman** | SalehAboufour  2022  **KSA** | Rawas  2023  **KSA** | Wazqar  2024  **KSA** | AlMuharraq 2024  **KSA** |
| --- | --- | --- | --- | --- | --- | --- | --- | --- | --- |
| Nationality status or Language or marital status | Arabic speaking: Yes (n = 58, 13%); No (387, 87%) | Indian (44.5%); Filipino (41.0%); Arabic cultures (11.73%; 78.6% of which are Saudi nationals); Western and other Asian cultures (2.74%) | ____ | Saudi: 63 (17.9%)/ Indian: 136 (38.7%)/ Filipino: 152 (43.3%) | Omani: 50 (18.5%)/ Non-Omani: 220 (81.5%) | ____ | Saudi (n = 35, 19 %); Non-Saudi (n = 149, 81%) | Single: 28(27.7)/ Married: 71 (70.3%)/ Separated/divorced: 2 (2.0%) | Saudi = 349(86.8%); Non-Saudi = 53 (13.2%) |
| Gender distribution | Male: 55 (12.4%)/ Female: 389 (87.4%) | ____ | Male: 10.4%/ Female: 89.6% | Male: 30 (8.5%)/ Female: 321 (91.5%) | Male:38 (14.1%)/ Female: 232 (85.9%) | ____ | Male: 32 (17.4%)/ Female: 152 (82.6%) | Male: 40 (39.6%)/ Female: 61 (60.4%) | M = 64(15.9%); F = 338 (84.1%) |
| Mean age (years) ± SD | 20-30 years: 141 (31.7%)/ 31-40 years: 182 (40.9%)/ 41-50 years: 76 (17.1%)/ 51-60 years: 46 (10.3%)  M ≈ 35.11 | ____ | Mean= 35 years/ SD= 8.25 | Mean= 28.63/ SD= 4.77 | Mean: 33.06/ SD: 5.82 | ____ | 20-29: 71(38.6%)/ 30-39: 86 (46.7%)/ 40-49: 22 (12.0%)/ 50 and more: 5 (2.7%) M ≈ 32.5 | Mean: 31 years/ SD: (2.35%) | 18-30 = 220 (54.7%); 31-45 = 172 (42.8%); >46 = 10 (2.5%) M ≈ 30.8 |
| Nurses' classification Or units | Staff nurse: 373 (83.8%)/ charge nurse: 44 (9.9%)/ head nurse: 10 (2.2)/ educator: 13 (2.9%)/ other: 3(0.7%) | ____ | surgical wards (30%), intensive care units (29%), medical wards (16.7%), obstetrics units (13%, paediatrics wards (6.3%) and non-specific units (5.1%) | Staff nurse (n = 323, 92%); Nurse with administrative functions (n = 28, 8%)& Different units: 28 (8.0%)/ Medicine: 60 (17.1%)/ Surgery: 70 (19.9%)/ Obstetrics: 65 (18.5%)/ Paediatrics: 42 (12.0%)/ Emergency department: 38 (10.8%)/ ICU: 34 (13.7%) | Paediatric ICU: 55 (20.4%)/ NICU:45 (16.7%)/ Adult ICU: 134 (49.6%)/ Cardiac ICU: 8 (3%)/ CCU: 28 (10.4%) | Nurses (n = 221, 28.3%) | Medical (n = 52, 28.3%); Surgical (n = 52, 28.3%); Obstetrics (n = 12, 6.5%); Pediatrics (n = 15, 8.2%); Oncology (n = 9, 4.9%); ICU (n = 44, 23.9) | Oncology working area: Medical (n = 33, 32.7%); Hematology (n = 19, 18.8%); Surgical (n = 5, 5%); Palliative (n = 20, 19.8%); Chemotherapy (n = 24, 23.7%) | ____ |
| Length of experience | Years in Nursing profession 1-5 years (n = 85, 19.1%); 6-10 years (n = 139, 31.2%); 11-15 years (n = 107, 24%); 16-20 years (n = 51, 11.5%); 21 years or more (n = 62, 13.9%). Length of time working at current hospital: Less than 1 year (n = 7, 1.6%); More than one year to two years (n = 109, 24.5%); More than two years to five years (n = 240, 53.9%); More than five years (n = 89, 20%) | ____ | In nursing profession Mean= 12.6 years/ SD= 8.03 | Years in current hospital <1 (n = 87, 24.8%); 1-5 (n = 181, 51.6%); 6-10 (n = 60, 17.1%); >/= 11 (n = 23, 6.6%). Years current area/unit: <1 (n = 93, 26.5%); 1-5 (n = 202, 57.5%); 6-10 (n = 44, 12.5%); >/= 11 (n = 12, 3.4%). Years in profession: <1: 53 (15.1%)/ 1-5: 200 (57.0%)/ 6-10: 74(21.1%)/ 11 or more: 24 (6.8%) | experience in your unit: Mean= 7.01/ SD= 5.05 | ____ | In nursing profession >1 year: 23 (12.5%)/ 1-5: 82 (44.6%)/ 6-10: 45 (24.5%)/ 11-15: 24 (13.0%)/ 16-20: 6 (3.3%)/ 21 or more:4 (2.2%) | Professional experience: </= 5 (n = 36, 35.6%); 6-15 (n = 62, 61.4%); >15 (n = 3, 3%). Unit experience: less than 1 year (n = 3, 3%); 1-5 years (n = 31, 30.7%); 6-10 years (n = 61, 60.4%); 11-15 years (n = 4, 4%); 16-20 years (n = 2, 2%) | 0-10 = 295 (73.4%); 11-25 = 104 (25.9%); >25 = 3(0.7%) |
| Level of education | Associates degree: 38 (8.5%)/ Bachelor degree: 380 (85.4%)/ Master degree: 14 (3.1%)/ Doctoral degree: 1 (0.2%) | ____ | Diploma (65.4%); Baccalaureate (34.6%) | Diploma: 24 (6.8%)/ Bachelor: 312 (88.9%)/ Master: 15 (4.3%) | Diploma: 90 (33.3%)/ Bachelor: 169 (62.6%)/ Postgraduate: 11 (4.1%) | ____ | Diploma: 14 (7.6%)/ Bachelor: 167 (90.8%) / Master: 3 (1.6%) | Diploma: 5 (4.9)/ Bachelor: 85 (84.2%)/ Master, PhD: 11 (10.9%) | Diploma = 138 (34.3%); Bachelor = 241 (60%); Master = 23 (5.7%) |
| Shift time or work hours | Day: 209 (47%)/ Night: 37 (8.3%)/ Day/Night: 196 (44%) | ____ | ____ | Work hours per week: <40 hours: 22 (6.3%)/ 40-59: 288 (82.1%)/ 60 or more: 41 (11.7%) | Working hours: Mean= 36.84/ SD= 3.19 | ____ | Working hours per week: 20 - 39 (n = 10, 5.4%); 40 to 59 (n = 124, 67.4%)/  60 - 79 (n = 50, 27.1%) | Weekly work time (h): </= 40 (n = 21 (20.8%); > 40 (n = 80, 79.2%) | ____ |
